# Granular Insights on Innate and Intrinsic Immunity to Flaviviruses

**DOI:** 10.3390/microorganisms13092091

**Published:** 2025-09-08

**Authors:** Janine Hvizdos, Alex C. Hofler, Shelton S. Bradrick

**Affiliations:** Trudeau Institute, Saranac Lake, NY 12983, USA; j9hvizdos@gmail.com (J.H.); ahofler@trudeauinstitute.org (A.C.H.)

**Keywords:** Zika virus, flavivirus, *Flaviviridae*, stress granules, eIF2α, RNA translation

## Abstract

Interaction between pathogenic human RNA viruses and host stress granules is an active area of research. Understanding how viruses manipulate, evade, and/or parasitize stress granules and related assemblies may lead to novel approaches for therapeutic and vaccine development. However, knowledge gaps remain, and the field is laden with conflicting conclusions. Stress granules have been implicated to serve as hubs for antiviral signaling pathways, thereby serving to indirectly restrict virus infection through enhancing innate immune responses. More recent evidence suggests that stress granules can exert intrinsic anti-viral properties through direct sequestration of viral RNAs without impacting immune signaling. Here we critically review the literature relevant to specific members of the *Flaviviridae* with particular focus on Zika virus.

## 1. Introduction

Innate immunity is the most fundamental form of protection against invading pathogens, operating via various mechanisms in most organisms. In contrast, adaptive immune functions tailored to specific pathogens are restricted to vertebrate species. A traditional view of the vertebrate innate immune system entails, in part, direct action by myeloid cells and humoral effectors on foreign invaders [1]. For example, neutrophils are highly effective at detecting and eliminating bacteria whereas the humoral complement system can promote chemotaxis and inflammation. Once activated, innate immune cells present antigens and supply critical extracellular cytokine signals that enable development of antigen-specific antibody and T cell-mediated responses, allowing for the adaptive immune system to target and destroy invading pathogens with specificity [2]. 

With respect to viral innate immunity, intracellular receptors have been well characterized to recognize non-self, viral molecular patterns, leading to induction of signal transduction pathways that trigger expression of anti-viral cytokines. Classic examples include activation of retinoic acid-inducible gene I (RIG-I) and toll-like receptor 3 (TLR3) by foreign viral RNA features, ultimately leading to the transcriptional induction of the key antiviral cytokine, interferon-β (IFN-β), and other pro-inflammatory genes [3,4]. IFN-β and other type I (IFN-α) and III (IFN-λ) IFNs induce expression of so-called interferon-stimulated genes (ISGs) that act to limit virus infection [5]. 

Less well characterized are constitutively expressed intracellular factors that act directly to limit virus infection independently of antiviral cytokines, referred to as intrinsic restriction factors [6]. This review focuses on phase-separated cytoplasmic granules [7] and their emerging roles in the intrinsic control of virus infection. We further focus on members of the flavivirus family, particularly Zika virus (ZIKV), and their interactions with cytoplasmic structures epitomized by stress granules. The relationship between the *Flaviviridae* and cellular stress was comprehensively reviewed by Valadão et al. in 2016 [8]. However, additional relevant studies have been conducted on viruses in this family in the last ~9 years, highlighting the need for further analysis. Specifically, we review works that contradict the earlier immune activation hypothesis involving stress granules and speculate on the contemporary hypothesis of viral RNA sequestration by stress granules, as well as subgenomic flavivirus RNAs and their potential role in subverting stress granule formation. To identify relevant articles for inclusion in this review, searchable databases (PubMed, Google Scholar) were used with search terms “flavivirus” and “stress granules”. The same databases were used to identify relevant background articles.

## 2. What Are Stress Granules?

Stress granules (SGs) are a species of RNA/protein condensates induced by various stimuli, and their existence spans kingdoms of life [9]. They contain mRNAs associated with stalled translational pre-initiation complexes (PICs), which are composed of initiation factors and small (40S) ribosomal subunits, and are further condensed with a variety other RNA-binding proteins (RBPs; G3BP1/2, TIAR, and Tia-1, among many others) within the cytoplasm [10]. Notably, G3BP1 is a critical core component of SGs and has been reported to be essential for SG formation [11]. SGs are liquid phase and dynamic [12,13,14,15], with both mRNAs and proteins moving continuously between phase-separated SGs and the cytoplasm. SGs themselves may persist for hours while the residence times of mRNAs within SGs are measured in seconds. Intriguingly, SGs have recently been reported to serve a structural role for mitigating endomembrane damage, serving as physical “plugs” of ruptures in endolysosomes [16]. SGs may also serve a triage function of mRNA, sorting transcripts to be directed toward reinitiation of translation or degradation, which may explain their involvement in the antiviral response.

## 3. What Causes SGs to Assemble?

SG formation is believed to require generalized translational arrest associated with the integrated stress response [17]. Specifically, they form when translation is stalled at the initiation phase. Evidence suggests that once initiation is complete and translational elongation has commenced, actively translated mRNAs are no longer subject to condensation into SGs until after ribosome(s) terminate and dissociate [18,19]. Large (60S) ribosomal subunits do not localize in SGs whereas small (40S) subunits and associated initiation factor proteins are readily detectable [20,21]. Stabilization of polyribosomes antagonizes SG formation, while treatments that promote polysome disassembly are compatible with and sometimes promote SG formation. Accordingly, the drugs emetine and cycloheximide, which stall elongation by trapping assembled 80S ribosomes on mRNA, simultaneously inhibit translation and stress granule formation. Conversely, puromycin, which inhibits translation by promoting premature termination and polysome disassembly, enhances SG formation [13,19].

Disruption of translational initiation is frequently due to the activity of stress-responsive protein kinases which phosphorylate the alpha subunit of eIF2 (eIF2α) on serine 51, thereby inhibiting the guanine nucleotide exchange function of eIF2B, leading to depletion of critical initiator tRNA ternary complexes (eIF2-GTP-Met-tRNA_i_) [22]. The stalling of initiation results in polysome disassembly as ribosomes in the elongation stage of translation run off transcripts and no new ribosomes take their place. Transcripts associated with a stalled PIC and lacking elongating ribosomes are subject to condensation into SGs [21] though it should be noted that ~90% of bulk mRNA, while translationally repressed under stress conditions, has been reported to remain outside of SGs [23]. Moreover, specific transcripts are either preferentially enriched or excluded from SGs, potentially due to inherently different translational efficiencies and/or mRNA lengths with shorter and efficiently translated mRNAs relatively resistant to SG association [24,25,26].

There are five identified stress-responsive kinases which phosphorylate eIF2α in response to disparate stressful stimuli [27]. These include (i) HRI [28], activated by heme deficiency to prevent excess globin translation in iron-deficient erythroid cells, (ii) PERK [29], activated by endoplasmic reticulum (ER) stress and heat/cold shock, (iii) GCN2 [30], activated by starvation and UV damage, (iv) MARK2 [31], activated by proteotoxic stress, and (v) PKR [32], activated by viral infection through sensing of double strand (ds)RNA. It should be noted that PKR is not the only eIF2α kinase that can respond to viral infection, as viruses impose more than one type of stress on the cell. For example, dengue virus [33] (DENV) and West Nile virus [34] (WNV) have been shown to activate PERK-mediated phosphorylation of eIF2α by inducing ER stress since the protein folding capacity of the ER can be overwhelmed by the influx of viral proteins during infection. While distinct stressors activate different kinases, all converge on the critical phosphorylation of Ser51-eIF2α [35] to effectively block translational initiation.

Inhibition of translation initiation by mechanisms independent of eIF2α phosphorylation can also drive SG formation. For example, inhibition of the mammalian target of rapamycin (mTOR) in selenium treated cells leads to activation of eIF4E-binding protein 1 (4E-BP1) which sequesters the critical cap-binding protein eIF4E. eIF4E is required for cap-dependent translation initiation and thus, its sequestration causes induction of non-canonical SGs [36]. Chemical inhibitors of translational initiation (e.g., pateamine A and hippuristanol) also promote the formation of SGs in an eIF2α-independent manner [37,38]. Of note, pateamine A was reported to trigger SGs through modulation of the eIF4A helicase that is both essential for translation initiation of most mRNAs and antagonizing RNA condensation [39].

## 4. What Happens to mRNAs in SGs?

There are at least three fates of mRNAs sequestered in SGs. First, resolution of stress may lead to SG disassembly and re-entry of silenced transcripts into the actively translated pool. Second, there is evidence that trapped mRNAs may be transferred to processing (P) bodies for storage or degradation [40,41]. Like SGs, P-bodies are phase-separated granular structures; however, they concentrate factors involved in RNA decay [42]. Lastly, SGs may be cleared by the cellular autophagy pathway, leading to destruction and recycling of SG mRNA and protein factors [43,44]. It should be noted that mRNA association with SG structures is reportedly transient even in the face of ongoing translational suppression [13,15].

## 5. Are SGs Antiviral?

It has been amply demonstrated that blocking translational initiation leads to SG formation, provided the necessary RBPs and mRNAs are present. It is less clear what the functions of the SGs might be in the context of virus infection, though evidence indicates that many viruses have evolved strategies to regulate SG formation [45]. Moreover, distinct granules triggered by activation of the host protein RNase L, an antiviral effector protein that degrades host and viral RNA, have been recently described [46]. These granules do not require translational suppression but depend on activation of RNase L by 2′-5′ oligoadenylates which are in turn synthesized by oligoadenylate synthetase, a well-established ISG [47].

Many viruses, including flaviviruses, have evolved means of blocking or reversing SG formation, frequently by cleaving or inhibiting the function of G3BP proteins or blocking eIF2α phosphorylation. This implies that there is a selective advantage for viruses to block SG assembly which, in turn, implies that SGs antagonize viral replication. Indeed, pioneering work by the Lloyd laboratory showed that when poliovirus, which cleaves G3BP1/2 and inhibits SG formation, is faced with a cleavage-resistant G3BP1, SG formation was rescued and virus replication was significantly reduced [48].

But do SGs serve direct antiviral function(s)? And if so, how? There are at least two, non-mutually exclusive hypotheses to explain the putative antiviral function of SGs: (i) promotion of IFN expression via enhanced activation of innate immune signaling pathways, and/or (ii) direct sequestration of viral transcripts and/or genomes from host translation machinery or sites of viral RNA synthesis. The latter hypothesis may be considered a form of intrinsic immunity that invokes SGs as direct-acting antiviral assemblies.

## 6. The Immune Activation Hypothesis

Studies have suggested that SGs play a role in activating the RIG-I-like receptor-mitochondrial antiviral signaling–interferon regulatory factor 3 (RLR-MAVS-IRF3) pathway [49]. It has been hypothesized that SGs concentrate RNA with viral signatures (dsRNA and/or 5′-triphosphorylated-RNA) in proximity to pattern recognition receptors such as MDA-5 and RIG-I, resulting in the activation of the MAVS signaling complex, the phosphorylation and nuclear translocation of the IRF3 transcription factor, and the expression of IFN-β and other transcriptional targets [50,51]. However, it is difficult to imagine how viral dsRNA and/or uncapped 5′-ppp-RNAs might become associated with stalled 43S PICs in SGs as they are generally believed to not be efficient substrates for translation initiation [52]. One possibility is that granular structures containing viral RNA species and host sensor proteins are distinct from conventional SGs [53]. G3BP1 silencing in HeLa cells was reported to impair both SG formation and IFN-β expression activation [54]. Moreover, G3BP1 has been implicated to bind both viral dsRNA and RIG-I, promoting IFN responses [55,56]. However, whether these findings indicate a direct role for SGs per se in promoting innate immune responses has not been conclusively established, and this is further complicated by the emerging realization of diversity in cytoplasmic granule form and function.

In opposition to the immune activation hypothesis, Langereis et al. reported that, while MDA5 localizes to SGs, blocking SG formation through PKR depletion had no effect on the type I IFN response [57]. The authors concluded that the localization of MDA5 to SGs is not essential for induction of IFN-β transcription. Recent publications cast additional doubt on the SG-immune activation hypothesis. In A549 cells challenged with the poly(I:C) dsRNA mimic, leading to formation of SGs, G3BP1 failed to colocalize with RIG-I, MAVS, and IRF3 [58]. A slight colocalization of MDA-5 within SGs was observed, but G3BP1/2 knockout which ablates SG formation had no effect on downstream signaling events such as IRF3 phosphorylation, nuclear translocation, or the expression of IFN-β. Additional experiments were performed in the context of flavivirus infection with similar outcomes: both MAVS and IRF3 failed to colocalize with G3BP1 in the context of WNV and ZIKV infection, and G3BP1 knockout failed to alter IRF3 phosphorylation in WNV-infected cells or nuclear translocation in WNV and ZIKV-infected cells. The authors concluded that SGs do not alter RLR-MAVS-IRF3 signaling in response to dsRNA or flavivirus infection. In the case of yellow fever virus (YFV) infection, SGs were reported not to contribute to cytokine responses mediated by RIG-I and PKR [59]. Adding to the discrepant results, a recent report described a restraining role for SGs in RLR and PKR signaling, providing a “shock absorber” to prevent overactivation of innate immune responses and protecting cells from apoptosis [60].

It is difficult to draw concrete conclusions from these contrasting data produced by different studies. One cause of confounding results may be the lack of a strict definition for what constitutes an SG. For example, the presence of G3BP1 in cytoplasmic granular structures is typically accepted as evidence for SGs. However, it is possible that a variety of related but distinct cytoplasmic granular structures contain G3BP1. In addition, the use of different cell lines, stimulation conditions, infection time points or even SG marker antibodies that recognize different epitopes could influence experimental outcomes. Thus, different studies may not be evaluating apples to apples when analyzing so-called canonical SGs.

## 7. The Viral RNA Sequestration Hypothesis

A viral RNA that is condensed in a SG is predicted to be, at least temporarily, unavailable for translation and replication. Thus, sequestration of viral genomes and/or mRNA has the potential to directly interfere with the viral life cycle. It has been reported that SGs are enriched with longer RNAs, suggesting that long RNA transcripts are more susceptible to condensation and/or are slower to diffuse out of SGs than typical cellular mRNAs [24,61]. Some viral RNAs are indeed very long. Coronavirus genomes are the largest of the positive-strand RNA viruses at ~30 kb in length. Flavivirus genomes are, on average ~11 kb whereas even small RNA viruses of the *Picornaviridae* are ~7 kb. If viral RNAs are disproportionately recruited into SGs, this could negatively impact viral replication, especially if they are subsequently dispatched to P bodies for degradation, or if viral RNA-containing SGs are subject to autophagy.

Only recently has direct evidence been reported to support this hypothesis. Burke and colleagues found that disrupting the interaction between the SARS-CoV-2 N protein and G3BP1, which disrupts SG formation [62], leads to accumulation of viral RNA in so called viral aggregated RNA condensates (VARCs) [58]. Interestingly, this observation depended on the overall capacity for cells to support viral translation. Cells that stochastically displayed reduced viral translation, or cells treated with translation initiation inhibitors, show higher rates of VARCs which correlated with reduced viral burden. Burke et al. also examined WNV and ZIKV and found that, under normal conditions, infected cells containing SGs did not show VARCs (see ZIKV section below). However, artificially dampening translation by treating cells with pateamine A or hippuristanol lead to viral RNA condensation in VARCs. Together, these findings imply that SGs, or some variant(s) of phase-separated granules, can directly segregate viral genomes and are therefore predicted to be inherently antiviral [58]. Future studies that might correlate viral pathogenicity with VARC frequency, using naturally or artificially attenuated virus strains, might provide strong evidence for the biological relevance of VARCs to control RNA virus infection.

## 8. Stress Granules and Flavivirus Infections

We dedicate the remaining text to critical analysis of reported interactions between SGs and members of the *Flaviviridae*, focusing on ZIKV in particular. This topic was comprehensively reviewed by Valadao et al. in 2016 [8]. With the subsequent publication of multiple newer studies concerning SGs and flaviviruses, this area merits a fresh look.

## 9. West Nile Virus (WNV)

WNV is an encephalitic mosquito-borne flavivirus transmitted by Culex mosquitos. Although humans are considered a dead-end host, WNV infection can cause serious neurological disease which may be fatal or lead to long-term sequelae [63]. Like most other flaviviruses, there are no available vaccines or therapeutics to combat WNV infections.

The relevance of SGs to flavivirus infection was first reported by Margo Brinton’s group who observed that the SG proteins, TIA-1 and TIAR, colocalized with NS3, a viral protein essential for replication, in both WNV and DENV2-infected BHK-21 cells [64]. Moreover, at 24 h post-infection cells infected with either virus failed to produce SGs or efficiently phosphorylate eIF2α after 30 min treatment with arsenite, a commonly used SG inducer which activates the HRI kinase [65]. These results imply that the two flaviviruses under study actively inhibit eIF-2α phosphorylation and/or sequester TIAR which has been described as essential for SG formation [66]. This consequently blocks the assembly of SGs in infected cells. Interestingly, a follow up study by the same group reported that strains of a specific WNV lineage do induce SGs in ~30% of infected BHK-21 cells even at late times (36 h) post-infection [67]. This was correlated with high level synthesis of viral RNA early during infection, possibly triggering SG induction through activation of PKR. The most recent relevant study from the Brinton laboratory reported that WNV interferes with arsenite-induced SG formation, but not SGs stimulated by heat-shock or dithiothreitol treatment, due to elevated levels of cellular glutathione in infected cells which neutralizes the ability of arsenite to drive reactive oxygen species and, consequently, HRI phosphorylation of eIF2α [68].

## 10. Hepatitis C Virus (HCV)

HCV is a member of the *Flaviviridae* and sole constituent of the hepacivirus genome that differs from arthropod-borne flaviviruses in several important ways [69]. It establishes a chronic infection in the majority of exposures and is transmitted via the blood-borne route. Chronic infection may lead to development of liver cirrhosis and hepatocellular carcinoma. The HCV RNA genome is uncapped, lacks a 3′ poly(A) tail, and contains an internal ribosome entry site (IRES) that drives translation initiation by an unconventional mechanism that is believed to be distinct from capped flavivirus genomes [70], though this has been challenged [71]. Pertinent to this review, several studies have investigated the relevance of SGs to HCV infection. Building on prior research showing that HCV-infected cells display SGs at a low frequency [72], Ruggieri and colleagues reported that HCV infected Huh-7 cells treated with IFN-α showed relatively high levels (40% of cells) of SG formation compared to mock-treated/infected cells and IFN-treated/uninfected cells [14]. Importantly, these authors demonstrated transient SG induction and dissolution in infected and IFN-treated cells using live-cell imaging over 72 h, revealing that ~97% of cells displayed SGs at least once over the analysis period. This finding indicates that SGs oscillate and that standard “snapshot” analysis of SGs in fixed cells may underestimate the extent of the stress response in cultured cells. Ruggieri et al. went on to report that SG formation was PKR-dependent, associated with cellular translation repression (but not viral replication burden) and that SG oscillation was a feature of multiple other virus infections. Notably, relatively few Huh-7 cells infected with DENV-2 (New Guinea C strain) showed SG induction.

Several other notable studies have been performed on HCV. Work from the Chisari laboratory suggested that HCV-induced SGs act to limit the translational efficiency of ISG mRNAs which may promote viral infection and persistence [73]. Optimal HCV infection also depended on several SG components, including G3BP1, in agreement with previous reports [74,75]. This was determined by RNA interference (RNAi)-mediated knockdown experiments which suggested SG proteins act, at least in part, at late stage(s) of infection. In contrast, Pager et al. reported that G3BP1 depletion by RNAi modestly reduced cell-associated viral RNA but increased levels of secreted infectious virus, suggesting that G3BP1 interfered with a late step of the HCV life cycle [76]. Notwithstanding the varying results, the relatively mild effects of G3BP1 knockdown on HCV infection suggest that it is a peripheral host factor and that SG induction during HCV infection does not substantially benefit or limit the virus in an in vitro setting.

## 11. Tick-Borne Flaviviruses

Tick-borne encephalitis virus (TBEV) and Powassan virus (POWV) are transmitted via ticks which have a much longer life span than mosquitos. As a result, tick-borne flaviviruses must persist for longer periods in their vector host than mosquito-borne viruses. This may be associated with reduced genomic variation in these viruses compared to mosquito-borne flaviviruses. Like WNV, both TBEV and POWV are encephalitic, and infection may lead to fatal outcome.

Albornoz et al. [77] used several imaging approaches to interrogate TBEV interactions with TIA-1 and TIAR: (i) cells expressing MS2 coat-protein were infected with a TBEV replicon containing an array of MS2 binding sites, allowing viral genomes to be fluorescently labeled using antibody directed against MS2, (ii) staining cells with polyclonal TBEV anti-serum to image viral proteins, and (iii) staining cells with anti-double strand RNA antibody. Using these three systems, the authors showed that TBEV RNA and protein colocalize with TIA-1, suggesting that TIA-1 is sequestered to sites of viral replication. They subsequently showed that TIAR localized with viral dsRNA. Following these experiments, they questioned whether viral factors localized to SGs, and further immunostaining of G3BP1, eIF3, and eIF4B showed that, while SGs formed during the infection, TBEV proteins did not show co-localization. Finally, functional studies indicated that TIA-1 acts as a restriction factor for TBEV. How this might relate to SGs direct or indirect impact on TBEV infection remains unknown.

Work by Goonawardane et al. [78] illustrated how inconsistent data may stem from strain-to-strain variation. These authors used TBEV strain Vasilchenko (Vs), which has minimal cytopathic effects and strain Hypr, which is highly cytopathic. Also using replicons, the authors replaced the structural proteins of these strains with a Spinach2 aptamer, which fluoresces in the presence of a substrate to label viral RNA, followed by a luciferase gene to measure TBEV burden. When mammalian cells were infected with either the Hypr or Vs replicon, perinuclear granules formed, consistent with Albornoz et al. [77] Interestingly, in mammalian cells Hypr infection was more robust than Vs, while in insect cells Hypr and Vs showed similar levels of viral burden. Since these replicons were observed to form granules, the authors investigated the localization of viral RNA with SG proteins. Both Hypr and Vs replicons co-localized with TIAR; in contrast, only Vs localized with G3BP1. This is intriguing because Albornoz et al. utilized TBEV strain Neudoerfl, which is an attenuated strain similar to Vs. Gooawardane et al. proceeded to show that Hypr infection induced phosphorylation of IRF-3 and expression of both caspase-3 and -8, while Vs infection induced phosphorylation of AKT. These results show that strain-to-strain variation within the same virus species can be sufficient to induce differential stress responses.

## 12. ZIKV

ZIKV is a flavivirus of significant interest for multiple reasons, including its capacity for explosive spread in a naïve population as seen in the 2015–2016 American epidemic, its ability to transmit vertically and cause birth defects, and its ability to be sexually transmitted [79]. At the molecular level, ZIKV resembles other mosquito-borne flaviviruses in genome organization and virion structure. The biological basis for ZIKV’s unique pathogenesis features is not well understood.

**Figure 1:**
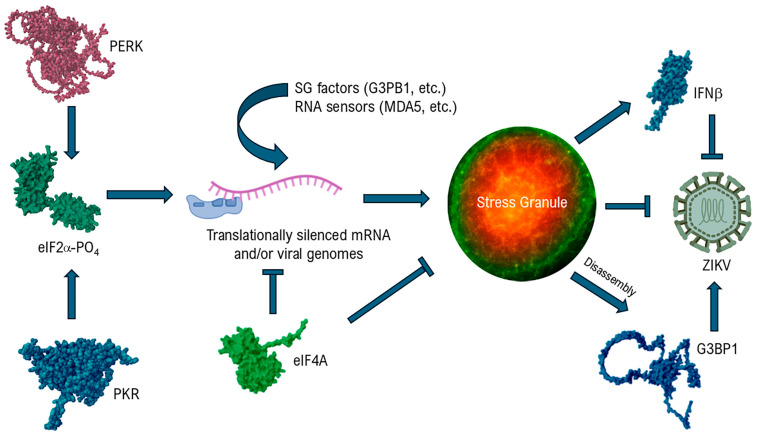
Factors in stress granule (SG) assembly and Zika virus (ZIKV) infection. Both protein kinase R (PKR) and PKR-like endoplasmic reticulum kinase (PERK) may be activated during ZIKV infection to trigger phosphorylation of eukaryotic initiation factor 2α (eIF2α). This event depletes ternary complex which causes initiating 43S pre-initiation complexes to stall on RNAs. Integration of translationally inactive viral or host ribonucleoprotein complexes with SG factors (G3BP1 and others) leads to SG assembly and sequestration of viral genomes. The RNA helicase and initiation factor, eIF4A, counteracts ribosome stalling and SG formation. SGs may enhance signaling to produce type I inteferons (IFN-β). Disassembled SG components may serve as host dependency factors for ZIKV and other flaviviruses. Protein structures shown were predicted by Alphafold 3 [99,100].

Table 1 and Figure 1 summarize the relevant studies on ZIKV discussed below. Roth and colleagues [80] initially reported that ZIKV (strain MR766) infected Huh-7 cells did not assemble SGs in response to arsenite treatment [81]. Interestingly, approximately 50% of infected cells that were not treated with arsenite displayed small granular structures that stained positive for eIF3B, TIAR, HuR and PCBP2, all proteins that localize to some extent in SGs [82]. Subsequent publications from Amorim et al. [83] and Hou et al. [84] in 2017 further addressed SGs in ZIKV infection. The former article showed that ZIKV (strain PRVABC59) infected Vero E6 cells were also relatively resistant to formation of arsenite-induced SGs: ~20% of infected cells were reported as SG positive compared to ~80% of uninfected cells. Use of pateamine A instead of arsenite showed that ZIKV did not interfere with SG formation when driven by altered eIF4A activity. On the other hand, arsenite or dithiothreitol treatment (which agonizes PERK) of ZIKV infected cells reduced, but did not eliminate, Ser-51 phosphorylation of eIF2α compared to uninfected cells. This observation suggests that ZIKV actively prevents eIF2α phosphorylation or accelerates the rate of eIF2α dephosphorylation. It is important to note that the effects reported are not absolute and that some fraction of infected cells appeared to be fully capable of inducing SGs (or variations thereof) upon arsenite treatment. Moreover, compared to control mock-infected cells, Vero E6 cells infected with ZIKV do appear to mount at least a muted stress response as indicated by relatively elevated levels (up to ~25-fold) of eIF2α Ser51 phosphorylation.

Hou and colleagues performed similar experiments as Amorim but used multiple virus strains and cell types [84]. Most experiments used the PLCal strain and human lung adenocarcinoma A549 cells or primary human fetal astrocytes (HFL). ZIKV-infected A549 cells showed fewer SGs per cell upon treatment with arsenite (an HRI agonist), poly(I:C) (a PKR agonist; delivered by transfection) or hippuristanol (an eIF4A modulator) compared to mock-infected cells, the latter result contrasting with data reported by Amorim et al. Moreover, ZIKV infection alone was sufficient to induce eIF2α phosphorylation at 24 and 48 h post-infection and infected cells treated with arsenite or transfected with poly(I:C) at 12 h post-infection displayed elevated levels of eIF2α phosphorylation compared to untreated cells. Correspondingly, Hou reported that ZIKV infection resulted in significant downregulation of cellular protein synthesis, especially late (48 h) in infection, in agreement with findings from Roth et al. [80]. Finally, Hou showed that multiple ZIKV proteins (especially capsid, NS3, and NS4A) could each independently reduce the average number of SGs in A549 cells treated hippuristanol. Taken together, these findings suggest that ZIKV impairs the infected cell’s capacity to induce SGs downstream of translational suppression, in contrast to conclusions reached by Amorim et al.

Work by Bonenfant and colleagues confirmed prior observations that ZIKV-infected cells are relatively resistant to arsenite-induced SG formation [85]. Interestingly, Huh-7 cells infected with different strains of ZIKV did show increased frequency (~10% of infected cells depending on SG marker used) of SGs compared to mock-treated cells, indicating that a low percentage of infected cells are capable of assembling SGs, in agreement with a recent study on ZIKV [86] and a 2015 study on DENV [87]. How these infected cells might differ from those that are resistant to forming SGs is not clear. Bonenfant et al. went on to characterize roles for selected SG components in virus infection using RNAi knockdown and overexpression and observed opposing effects of G3BP1 and HuR on ZIKV RNA replication: G3BP1 behaved as a host dependency factor while HuR acted as a host restriction factor. These authors also reported dramatic colocalization of viral double stranded RNA (dsRNA) with G3BP1 in infected cells, consistent with prior observations implicating reorganization of SG components to sites of virus replication [83,84]. This result implies that ZIKV-mediated sequestration of key SG proteins by an undefined mechanism may be responsible for inhibition of SG assembly in most infected cells.

Two recent studies further addressed SGs and ZIKV infection. Wu et al. provided evidence that ZIKV NS2B mediates eIF2α dephosphorylation by promoting interaction between eIF2α and the protein phosphatase 1α (PP1α), leading to prevention of translational suppression and SG assembly [88]. Unexpectedly, knockout of PP1α in HeLa cells actually resulted in enhanced ZIKV infection and complementation of knockout cells with PP1α reduced viral titers, likely through promoting type I IFN expression. Thus, any benefit that ZIKV gains through antagonizing host translation shutoff and SG assembly via PP1α modulation may be outweighed by an enhanced IFN response, at least in HeLa cells.

The second study from the laboratory of Roy Parker was described in part above [58] (see the section on viral RNA sequestration). These authors conducted cell level analysis of ZIKV infected A549 cells, examining viral RNA localization, SG status, and levels of phosphorylated eIF2α. It was observed that infected cells that were positive for SGs also showed prominent eIF2α phosphorylation. However, ZIKV RNA in these cells was excluded from SGs as marked by G3BP1, suggesting that ZIKV has evolved mechanism(s) that allow escape from translational repression arising from depleted ternary complex levels. Of note, arsenite treatment of ZIKV-infected cells lead to increased eIF2α phosphorylation and the number of infected cells containing SGs, in contrast to observations reported in previous studies [83,84,85].

How do we reconcile the apparently conflicting conclusions reached by different published articles? Unfortunately, this is not an easy question to answer with available data at the present time. As alluded to above, there is likely to be diversity of cytoplasmic granular structures that have divergent but overlapping functions in the context of virus infections, including SGs, P bodies, double stranded RNA-induced foci (dRIF), VARCs, and RNase L-induced bodies (RLBs) [61]. It is possible that granular structures deemed to be 100% SGs may in fact be a mixture of different types of granules depending on marker(s) used or not used. Even different methods for defining whether a cell is SG positive or not could introduce bias. Other important factors include cell type(s) used, IFN competence, multiplicity of infection, analysis methods, time points(s) selected for analysis, etc. What can be concluded is that interactions between members of the *Flaviviridae* and host phase separated granules are complex and likely involve multiple independent mechanisms operating simultaneously. Future well-controlled and rigorous studies are needed to clarify these mechanisms and define their relevance to diseases caused by this important family of viruses.

## 13. Could Flaviviruses Target SGs Using RNA?

Multiple flavivirus proteins have been implicated to suppress SG assembly via independent mechanisms [84,88,89]. Besides viral proteins, flavivirus infected cells accumulate subgenomic flavivirus RNAs (sfRNAs) due to incomplete exonucleolytic degradation of genomic RNA by the host 5′ to 3′ exonuclease, Xrn1 [90,91]. The resulting sfRNA corresponds to most of the genomic 3′ untranslated region and is highly structured. Many studies have documented pro-viral roles of sfRNAs in virus infection [92]. The mechanism of action is believed to entail “sponging” and consequent inhibition of antiviral factors by sfRNAs, though activation of PKR by the ZIKV sfRNA has recently been described to promote infection through suppressing translation of type I IFN and ISG mRNAs [86].

Could sfRNAs play a role in antagonizing SG assembly? The sfRNAs of DENV have been shown to bind multiple SG factors including DDX6, G3BP1, G3BP2 and caprin [93,94]. While this was described to limit translation of ISG mRNAs, it is possible that these interactions also impair SG assembly. A second clue was provided in a study by an author of this review [95]. Infection of HeLa cells with a mutant ZIKV that is defective for sfRNA production led to increased levels of cytoplasmic granular structures, as marked by the RNA-binding protein FXR2, compared to wild type ZIKV. As FXR2 has been described to localize to SGs [96], this observation provides genetic support to the hypothesis that ZIKV sfRNA antagonizes SG assembly. Notably, the ZIKV mutant tested is highly attenuated, in part due to reduced capacity for RNA synthesis, and serves as an effective live-attenuated vaccine strain in mice and rhesus macaques [97,98]. It is tempting to speculate that this engineered vaccine strain’s hypothetical inability to reduce SG assembly may contribute to the attenuated phenotype. Further research is needed to explore this possibility.

## Figures and Tables

**Table 1:** Detailed summary of ZIKV studies related to stress granules *.

Article	Year	Cell Type(s) Used	ZIKV Strains Used	Time Point(s) Analyzed	MOI	Treatments	Effects on SGs	Effects on eIF2α Phosphorylation
Roth et. al.	2017	Huh7	MR766	24 h.p.i	0.5	Naive or 0.5 mM arsenite for 45 min	ZIKV inhibited SG formation in arsenite treated cells; 55% of ZIKV-infected naive cells showed eIF3B positive foci	Not performed for ZIKV; No increase in phosphorylation of DENV-infected cells
Amorim et. al.	2017	Vero	PRVABC59	24 h.p.i	0.5	0.5 mMM arsenite for 1 h	ZIKV infected cells show reduced SG frequency (~20%) upon arsenite treatment compared to mock-infected cells (~80%)	No effect due to infection; ZIKV reduced stress-induced phosphorylation
					0.5	50 nM Pateamine A for 2 h	ZIKV infection did not inhibit Pateamine A-induced SG assembly	
					0.5	1 mM selenite for 2 h	ZIKV infection did not inhibit selenite-induced SG assembly	
Hou et. al.	2017	A549, primary HFAs, Huh7	MR766, PLCal	24, 48, 72 h.p.i	3	Naive	ZIKV strains do not induce significant SG formation in A549 or primary HFA cells.	Elevated phosphorylation due to infection
		A549, HFAs	Not indicated	24 (A549) or 48 (HFAs) h.p.i	3 (A549) or 5 (HFAs)	0.5 mM arsenite for 30 min	ZIKV inhibited SG formation in arsenite treated cells	
		A549	Not indicated	24 h.p.i	3	1 mM hippuristanol for 25 min	ZIKV inhibited SG formation in hippuristanol treated cells	
Bonenfant et. al.	2019	Huh7	Cambodia 160310, MR766, PRVABC59	24 h.p.i	5	1 mM arsenite for 30 min	All ZIKV strains tested inhibit SG formation in arsenite treated cells.	Not performed
Wu et. al.	2024	HeLa	Not indicated	12, 24, 36 h.p.i	0.3	0.2 mM arsenite for 1 h	ZIKV did not induce SG formation and inhibited formation induced by arsenite	No effect due to infection; ZIKV reduced stress-induced phosphorylation
			Not indicated	36 h.p.i		2 nM dithiothreitol for 1 h	ZIKV infected cells treated with dithiothreitol had a lower percentage of SG formation compared to mock	
			Not indicated	12 h.p.i		2 ng poly I:C for 12 h	ZIKV infected cells treated with poly I:C had a lower percentage of SG formation compared to mock	
		A549	Not indicated	24 h.p.i	0.1	0.5 mM arsenite for 1 h	ZIKV infected cells displayed decreased percentage of SG assembly compared to mock	
Burke et. al.	2024	A549-RL-KO	PB81	24 h.p.i.	10	Naive or 0.5 mM arsenite for 1h	~10% of ZIKV infected naive cells contained SGs; Arsenite increased frequency of SGs to >60% of infected cells	Elevated phosphorylation due to infection
						1 uM hippuristanol for 1 h	Increase in G3BP1 assemblies in SGs containing viral RNAs	
Pallares et. al.	2024	A549	ARCB116141	24 h.p.i.	5	Naive	ZIKV infected cells displayed higher percentage of SG accumulation compared to uninfected cells	Elevated phosphorylation due to infection

* Additional factors that may impact experimental results not listed in Table 1 include methodological variables, such as specific antibodies used for SG marker visualization and criteria for defining cells as positive for SGs, and cellular IFN competence.

## Data Availability

No new data were created or analyzed in this study.

## Abbreviations Used

4EBP	eIF4E-binding protein
DENV	dengue virus
dRIF	double stranded RNA-induced foci
dsRNA	double-strand RNA
eIF	eukaryotic initiation factor
ER	endoplasmic reticulum
HCV	hepatitis C virus
HFL	human fetal astrocytes
IFN	interferon
IRES	internal ribosome entry site
IRF	interferon regulatory factor
ISG	interferon-stimulated gene
MAVS	mitochondrial antiviral signaling
mTOR	mammalian target of rapamycin
PIC	pre-initiation complex
POWV	Powassan virus
PP	protein phosphatase
RIG-I	retinoic acid-inducible gene I
RLB	RNase L-induced bodies
RLR	RIG-I-like receptor
RNA	ribonucleic acid
RNAi	RNA interference
RNase	ribonuclease
sfRNA	subgenomic flavivirus RNA
SG	stress granule
TBEV	tick-born encephalitis virus
TBFV	tick-borne flavivirus
TLR	toll-like receptor
VARC	viral aggregated RNA condensates
WNV	West Nile virus
YFV	yellow fever virus
ZIKV	Zika virus

## References

[1] Beutler B. (2004). Innate immunity: An overview. Mol. Immunol..

[2] Iwasaki A., Medzhitov R. (2015). Control of adaptive immunity by the innate immune system. Nat. Immunol..

[3] Yoneyama M., Kato H., Fujita T. (2024). Physiological functions of RIG-I-like receptors. Immunity.

[4] Takeda K., Kaisho T., Akira S. (2003). Toll-like receptors. Annu. Rev. Immunol..

[5] Schoggins J.W. (2019). Interferon-Stimulated Genes: What Do They All Do?. Annu. Rev. Virol..

[6] Yan N., Chen Z.J. (2012). Intrinsic antiviral immunity. Nat. Immunol..

[7] Alberti S. (2017). Phase separation in biology. Curr. Biol..

[8] Valadão A.L., Aguiar R.S., de Arruda L.B. (2016). Interplay between Inflammation and Cellular Stress Triggered by Flaviviridae Viruses. Front. Microbiol..

[9] Anderson P., Kedersha N. (2009). Stress granules. Curr. Biol..

[10] Youn J.Y., Dyakov B.J.A., Zhang J., Knight J.D.R., Vernon R.M., Forman-Kay J.D., Gingras A.C. (2019). Properties of Stress Granule and P-Body Proteomes. Mol. Cell.

[11] Yang P., Mathieu C., Kolaitis R.M., Zhang P., Messing J., Yurtsever U., Yang Z., Wu J., Li Y., Pan Q. (2020). G3BP1 Is a Tunable Switch that Triggers Phase Separation to Assemble Stress Granules. Cell.

[12] Hofmann S., Kedersha N., Anderson P., Ivanov P. (2021). Molecular mechanisms of stress granule assembly and disassembly. Biochim. Biophys. Acta—Mol. Cell Res..

[13] Mollet S., Cougot N., Wilczynska A., Dautry F., Kress M., Bertrand E., Weil D. (2008). Translationally repressed mRNA transiently cycles through stress granules during stress. Mol. Biol. Cell.

[14] Ruggieri A., Dazert E., Metz P., Hofmann S., Bergeest J.P., Mazur J., Bankhead P., Hiet M.S., Kallis S., Alvisi G. (2012). Dynamic Oscillation of Translation and Stress Granule Formation Mark the Cellular Response to Virus Infection. Cell Host Microbe.

[15] Bley N., Lederer M., Pfalz B., Reinke C., Fuchs T., Glaß M., Möller B., Hüttelmaier S. (2015). Stress granules are dispensable for mRNA stabilization during cellular stress. Nucleic Acids Res..

[16] Bussi C., Mangiarotti A., Vanhille-Campos C., Aylan B., Pellegrino E., Athanasiadi N., Fearns A., Rodgers A., Franzmann T.M., Šarić A. (2023). Publisher Correction: Stress granules plug and stabilize damaged endolysosomal membranes. Nature.

[17] Pakos-Zebrucka K., Koryga I., Mnich K., Ljujic M., Samali A., Gorman A.M. (2016). The integrated stress response. EMBO Rep..

[18] Buchan J.R., Muhlrad D., Parker R. (2008). P bodies promote stress granule assembly in Saccharomyces cerevisiae. J. Cell Biol..

[19] Kimball S.R., Horetsky R.L., Ron D., Jefferson L.S., Harding H.P. (2003). Mammalian stress granules represent sites of accumulation of stalled translation initiation complexes. Am. J. Physiol. Cell Physiol..

[20] Fedorovskiy A.G., Burakov A.V., Terenin I.M., Bykov D.A., Lashkevich K.A., Popenko V.I., Makarova N.E., Sorokin I.I., Sukhinina A.P., Prassolov V.S. (2023). A Solitary Stalled 80S Ribosome Prevents mRNA Recruitment to Stress Granules. Biochemistry.

[21] Kedersha N., Chen S., Gilks N., Li W., Miller I.J., Stahl J., Anderson P. (2002). Evidence that ternary complex (eIF2-GTP-tRNA(i)(Met))-deficient preinitiation complexes are core constituents of mammalian stress granules. Mol. Biol. Cell.

[22] Bogorad A.M., Lin K.Y., Marintchev A. (2017). Novel mechanisms of eIF2B action and regulation by eIF2 phosphorylation. Nucleic Acids Res..

[23] Khong A., Matheny T., Jain S., Mitchell S.F., Wheeler J.R., Parker R. (2017). The Stress Granule Transcriptome Reveals Principles of mRNA Accumulation in Stress Granules. Mol. Cell..

[24] Curdy N., Lanvin O., Cerapio J.P., Pont F., Tosolini M., Sarot E., Valle C., Saint-Laurent N., Lhuillier E., Laurent C. (2023). The proteome and transcriptome of stress granules and P bodies during human T lymphocyte activation. Cell Rep..

[25] Van Leeuwen W., VanInsberghe M., Battich N., Salmén F., van Oudenaarden A., Rabouille C. (2022). Identification of the stress granule transcriptome via RNA-editing in single cells and in vivo. Cell Rep. Methods.

[26] Namkoong S., Ho A., Woo Y.M., Kwak H., Lee J.H. (2018). Systematic Characterization of Stress-Induced RNA Granulation. Mol Cell..

[27] Costa-Mattioli M., Walter P. (2020). The integrated stress response: From mechanism to disease. Science.

[28] Han A.P., Yu C., Lu L., Fujiwara Y., Browne C., Chin G., Fleming M., Leboulch P., Orkin S.H., Chen J.J. (2001). Heme-regulated eIF2alpha kinase (HRI) is required for translational regulation and survival of erythroid precursors in iron deficiency. EMBO J..

[29] Harding H.P., Zhang Y., Bertolotti A., Zeng H., Ron D. (2000). Perk is essential for translational regulation and cell survival during the unfolded protein response. Mol. Cell..

[30] Berlanga J.J., Santoyo J., De Haro C. (1999). Characterization of a mammalian homolog of the GCN2 eukaryotic initiation factor 2α kinase. Eur. J. Biochem..

[31] Lu Y.N., Kavianpour S., Zhang T., Zhang X., Nguyen D., Thombre R., He L., Wang J. (2021). MARK2 phosphorylates eIF2α in response to proteotoxic stress. PLoS Biol..

[32] García M.A., Gil J., Ventoso I., Guerra S., Domingo E., Rivas C., Esteban M. (2006). Impact of Protein Kinase PKR in Cell Biology: From Antiviral to Antiproliferative Action. Microbiol. Mol. Biol. Rev..

[33] Peña J., Harris E. (2011). Dengue virus modulates the unfolded protein response in a time-dependent manner. J. Biol. Chem..

[34] Medigeshi G.R., Lancaster A.M., Hirsch A.J., Briese T., Lipkin W.I., DeFilippis V., Früh K., Mason P.W., Nikolich-Zugich J., Nelson J.A. (2007). West Nile Virus Infection Activates the Unfolded Protein Response, Leading to CHOP Induction and Apoptosis. J. Virol..

[35] Merrick W.C., Pavitt G.D. (2018). Protein synthesis initiation in eukaryotic cells. Cold Spring Harb Perspect. Biol..

[36] Fujimura K., Sasaki A.T., Anderson P. (2012). Selenite targets eIF4E-binding protein-1 to inhibit translation initiation and induce the assembly of non-canonical stress granules. Nucleic Acids Res..

[37] Dang Y., Kedersha N., Low W.K., Romo D., Gorospe M., Kaufman R., Anderson P., Liu J.O. (2006). Eukaryotic initiation factor 2α-independent pathway of stress granule induction by the natural product pateamine A. J. Biol. Chem..

[38] Mazroui R., Sukarieh R., Bordeleau M.E., Kaufman R.J., Northcote P., Tanaka J., Gallouzi I., Pelletier J. (2006). Inhibition of ribosome recruitment induces stress granule formation independently of eukaryotic initiation factor 2alpha phosphorylation. Mol. Biol. Cell.

[39] Tauber D., Tauber G., Khong A., Van Treeck B., Pelletier J., Parker R. (2020). Modulation of RNA Condensation by the DEAD-Box Protein eIF4A. Cell.

[40] Kedersha N., Stoecklin G., Ayodele M., Yacono P., Lykke-Andersen J., Fitzler M.J., Scheuner D., Kaufman R.J., Golan D.E., Anderson P. (2005). Stress granules and processing bodies are dynamically linked sites of mRNP remodeling. J. Cell Biol..

[41] Hubstenberger A., Courel M., Bénard M., Souquere S., Ernoult-Lange M., Chouaib R., Yi Z., Morlot J.B., Munier A., Fradet M. (2017). P-Body Purification Reveals the Condensation of Repressed mRNA Regulons. Mol. Cell.

[42] Riggs C.L., Kedersha N., Ivanov P., Anderson P. (2020). Mammalian stress granules and P bodies at a glance. J. Cell. Sci..

[43] Buchan J.R., Kolaitis R.M., Taylor J.P., Parker R. (2013). Eukaryotic stress granules are cleared by autophagy and Cdc48/VCP function. Cell.

[44] Yang C., Wang Z., Kang Y., Yi Q., Wang T., Bai Y., Liu Y. (2023). Stress granule homeostasis is modulated by TRIM21-mediated ubiquitination of G3BP1 and autophagy-dependent elimination of stress granules. Autophagy.

[45] White J.P., Lloyd R.E. (2012). Regulation of stress granules in virus systems. Trends Microbiol..

[46] Burke J.M., Lester E.T., Tauber D., Parker R. (2020). RNase L promotes the formation of unique ribonucleoprotein granules distinct from stress granules. J. Biol. Chem..

[47] Silverman R.H. (2007). Viral Encounters with 2′,5′-Oligoadenylate Synthetase and RNase L during the Interferon Antiviral Response. J. Virol..

[48] White J.P., Cardenas A.M., Marissen W.E., Lloyd R.E. (2007). Inhibition of Cytoplasmic mRNA Stress Granule Formation by a Viral Proteinase. Cell Host Microbe.

[49] Onomoto K., Yoneyama M., Fung G., Kato H., Fujita T. (2014). Antiviral innate immunity and stress granule responses. Trends Immunol..

[50] Oh S.W., Onomoto K., Wakimoto M., Onoguchi K., Ishidate F., Fujiwara T., Yoneyama M., Kato H., Fujita T. (2016). Leader-Containing Uncapped Viral Transcript Activates RIG-I in Antiviral Stress Granules. PLoS Pathog..

[51] Onomoto K., Jogi M., Yoo J.S., Narita R., Morimoto S., Takemura A., Sambhara S., Kawaguchi A., Osari S., Nagata K. (2012). Critical role of an antiviral stress granule containing RIG-I and PKR in viral detection and innate immunity. PLoS ONE.

[52] Hershey J.W.B., Sonenberg N., Mathews M.B. (2019). Principles of Translational Control. Cold Spring Harb Perspect. Biol..

[53] Corbet G.A., Burke J.M., Bublitz G.R., Tay J.W., Parker R., Acosta-Alvear D., Wu H. (2022). dsRNA-induced condensation of antiviral proteins modulates PKR activity. Proc. Natl. Acad. Sci. USA.

[54] Reineke L.C., Lloyd R.E. (2015). The Stress Granule Protein G3BP1 Recruits Protein Kinase R To Promote Multiple Innate Immune Antiviral Responses. J. Virol..

[55] Yang W., Ru Y., Ren J., Bai J., Wei J., Fu S., Liu X., Li D., Zheng H. (2019). G3BP1 inhibits RNA virus replication by positively regulating RIG-I-mediated cellular antiviral response. Cell Death Dis..

[56] Kim S.S.Y., Sze L., Lam K.P. (2019). The stress granule protein G3BP1 binds viral dsRNA and RIG-I to enhance interferon-β response. J. Biol. Chem..

[57] Langereis M.A., Feng Q., van Kuppeveld F.J. (2013). MDA5 Localizes to Stress Granules, but This Localization Is Not Required for the Induction of Type I Interferon. J. Virol..

[58] Burke J.M., Ratnayake O.C., Watkins J.M., Perera R., Parker R. (2024). G3BP1-dependent condensation of translationally inactive viral RNAs antagonizes infection. Sci. Adv..

[59] Beauclair G., Streicher F., Chazal M., Bruni D., Lesage S., Gracias S., Bourgeau S., Sinigaglia L., Fujita T., Meurs E.F. (2020). Retinoic Acid Inducible Gene I and Protein Kinase R, but Not Stress Granules, Mediate the Proinflammatory Response to Yellow Fever Virus. J. Virol..

[60] Paget M., Cadena C., Ahmad S., Wang H.T., Jordan T.X., Kim E., Koo B., Lyons S.M., Ivanov P., tenOever B. (2023). Stress granules are shock absorbers that prevent excessive innate immune responses to dsRNA. Mol. Cell.

[61] Watkins J.M., Burke J.M. (2024). A closer look at mammalian antiviral condensates. Biochem. Soc. Trans..

[62] Yang Z., Johnson B.A., Meliopoulos V.A., Ju X., Zhang P., Hughes M.P., Wu J., Koreski K.P., Clary J.E., Chang T.C. (2024). Interaction between host G3BP and viral nucleocapsid protein regulates SARS-CoV-2 replication and pathogenicity. Cell Rep..

[63] Ostlund E.N., Andresen J.E., Andresen M. (2000). West Nile encephalitis. Vet. Clin. N. Am. Equine Pract..

[64] Emara M.M., Brinton M.A. (2007). Interaction of TIA-1/TIAR with West Nile and dengue virus products in infected cells interferes with stress granule formation and processing body assembly. Proc. Natl. Acad. Sci. USA.

[65] McEwen E., Kedersha N., Song B., Scheuner D., Gilks N., Han A., Chen J.J., Anderson P., Kaufman R.J. (2005). Heme-regulated inhibitor kinase-mediated phosphorylation of eukaryotic translation initiation factor 2 inhibits translation, induces stress granule formation, and mediates survival upon arsenite exposure. J. Biol. Chem..

[66] Kedersha N.L., Gupta M., Li W., Miller I., Anderson P. (1999). RNA-binding proteins TIA-1 and TIAR link the phosphorylation of eIF-2 alpha to the assembly of mammalian stress granules. J. Cell Biol..

[67] Courtney S.C., Scherbik S.V., Stockman B.M., Brinton M.A. (2012). West Nile Virus Infections Suppress Early Viral RNA Synthesis and Avoid Inducing the Cell Stress Granule Response. J. Virol..

[68] Basu M., Courtney S.C., Brinton M.A. (2017). Arsenite-induced stress granule formation is inhibited by elevated levels of reduced glutathione in West Nile virus-infected cells. PLoS Pathog..

[69] Moradpour D., Penin F., Rice C.M. (2007). Replication of hepatitis C virus. Nat. Rev. Microbiol..

[70] Tsukiyama-Kohara K., Iizuka N., Kohara M., Nomoto A. (1992). Internal ribosome entry site within hepatitis C virus RNA. J. Virol..

[71] Song Y., Mugavero J.A., Stauft C.B., Wimmer E. (2019). Dengue and zika virus 5’ untranslated regions harbor internal ribosomal entry site functions. MBio.

[72] Jones C.T., Catanese M.T., Law L.M.J., Khetani S.R., Syder A.J., Ploss A., Oh T.S., Schoggins J.W., MacDonald M.R., Bhatia S.N. (2010). Real-time imaging of hepatitis C virus infection using a fluorescent cell-based reporter system. Nat. Biotechnol..

[73] Garaigorta U., Heim M.H., Boyd B., Wieland S., Chisari F.V. (2012). Hepatitis C Virus (HCV) Induces Formation of Stress Granules Whose Proteins Regulate HCV RNA Replication and Virus Assembly and Egress. J. Virol..

[74] Ariumi Y., Kuroki M., Kushima Y., Osugi K., Hijikata M., Maki M., Ikeda M., Kato N. (2011). Hepatitis C Virus Hijacks P-Body and Stress Granule Components around Lipid Droplets. J. Virol..

[75] Yi Z., Pan T., Wu X., Song W., Wang S., Xu Y., Rice C.M., MacDonald M.R., Yuan Z. (2011). Hepatitis C Virus Co-Opts Ras-GTPase-Activating Protein-Binding Protein 1 for Its Genome Replication. J. Virol..

[76] Pager C.T., Schütz S., Abraham T.M., Luo G., Sarnow P. (2013). Modulation of hepatitis C virus RNA abundance and virus release by dispersion of processing bodies and enrichment of stress granules. Virology..

[77] Albornoz A., Carletti T., Corazza G., Marcello A. (2014). The Stress Granule Component TIA-1 Binds Tick-Borne Encephalitis Virus RNA and Is Recruited to Perinuclear Sites of Viral Replication To Inhibit Viral Translation. J. Virol..

[78] Goonawardane N., Upstone L., Harris M., Jones I.M. (2022). Identification of Host Factors Differentially Induced by Clinically Diverse Strains of Tick-Borne Encephalitis Virus. J. Virol..

[79] Aliota M.T., Bassit L., Bradrick S.S., Cox B., Garcia-Blanco M.A., Gavegnano C., Friedrich T.C., Golos T.G., Griffin D.E., Haddow A.D. (2017). Zika in the Americas, year 2: What have we learned? What gaps remain? A report from the Global Virus Network. Antivir. Res..

[80] Roth H., Magg V., Uch F., Mutz P., Klein P., Haneke K., Lohmann V., Bartenschlager R., Fackler O.T., Locker N. (2017). Flavivirus infection uncouples translation suppression from cellular stress responses. MBio.

[81] Lu L., Han A.P., Chen J.J. (2001). Translation initiation control by heme-regulated eukaryotic initiation factor 2alpha kinase in erythroid cells under cytoplasmic stresses. Mol. Cell Biol..

[82] Protter D.S.W., Parker R. (2016). Principles and Properties of Stress Granules. Trends Cell Biol..

[83] Amorim R., Temzi A., Griffin B.D., Mouland A.J. (2017). Zika virus inhibits eIF2α-dependent stress granule assembly. PLoS Negl. Trop. Dis..

[84] Hou S., Kumar A., Xu Z., Airo A.M., Stryapunina I., Wong C.P., Branton W., Tchesnokov E., Götte M., Power C. (2017). Zika Virus Hijacks Stress Granule Proteins and Modulates the Host Stress Response. J. Virol..

[85] Bonenfant G., Williams N., Netzband R., Schwarz M.C., Evans M.J., Pager C.T. (2019). Zika Virus Subverts Stress Granules To Promote and Restrict Viral Gene Expression. J. Virol..

[86] Pallarés H.M., González López Ledesma M.M., Oviedo-Rouco S., Castellano L.A., Costa Navarro G.S., Fernández-Alvarez A.J., D’Andreiz M.J., Aldas-Bulos V.D., Alvarez D.E., Bazzini A.A. (2024). Zika virus non-coding RNAs antagonize antiviral responses by PKR-mediated translational arrest. Nucleic Acids Res..

[87] Xia J., Chen X., Xu F., Wang Y., Shi Y., Li Y., He J., Zhang P. (2015). Dengue virus infection induces formation of G3BP1 granules in human lung epithelial cells. Arch. Virol..

[88] Wu X., Zhang L., Liu C., Cheng Q., Zhao W., Chen P., Qin Y., Chen M. (2024). The NS2B-PP1α-eIF2α axis: Inhibiting stress granule formation and Boosting Zika virus replication. PLoS Pathog..

[89] Katoh H., Okamoto T., Fukuhara T., Kambara H., Morita E., Mori Y., Kamitani W., Matsuura Y. (2013). Japanese Encephalitis Virus Core Protein Inhibits Stress Granule Formation through an Interaction with Caprin-1 and Facilitates Viral Propagation. J. Virol..

[90] Pijlman G.P., Funk A., Kondratieva N., Leung J., Torres S., van der Aa L., Liu W.J., Palmenberg A.C., Shi P.Y., Hall R.A. (2008). A Highly Structured, Nuclease-Resistant, Noncoding RNA Produced by Flaviviruses Is Required for Pathogenicity. Cell Host Microbe.

[91] Chapman E.G., Costantino D.A., Rabe J.L., Moon S.L., Wilusz J., Nix J.C., Kieft J.S. (2014). The structural basis of pathogenic subgenomic flavivirus RNA (sfRNA) production. Science.

[92] Slonchak A., Khromykh A.A. (2018). Subgenomic flaviviral RNAs: What do we know after the first decade of research. Antivir. Res..

[93] Ward A.M., Bidet K., Yinglin A., Ler S.G., Hogue K., Blackstock W., Gunaratne J., Garcia-Blanco M.A. (2011). Quantitative mass spectrometry of DENV-2 RNA-interacting proteins reveals that the DEAD-box RNA helicase DDX6 binds the DB1 and DB2 3’ UTR structures. RNA Biol..

[94] Bidet K., Garcia-Blanco M.A. (2014). Flaviviral RNAs: Weapons and targets in the war between virus and host. Biochem. J..

[95] Soto-Acosta R., Xie X., Shan C., Baker C.K., Shi P.Y., Rossi S.L., Garcia-Blanco M.A., Bradrick S. (2018). Fragile X mental retardation protein is a Zika virus restriction factor that is antagonized by subgenomic flaviviral RNA. Elife.

[96] Markmiller S., Soltanieh S., Server K.L., Mak R., Jin W., Fang M.Y., Luo E.C., Krach F., Yang D., Sen A. (2018). Context-Dependent and Disease-Specific Diversity in Protein Interactions within Stress Granules. Cell.

[97] Shan C., Muruato A.E., Nunes B.T.D., Luo H., Xie X., Medeiros D.B.A., Wakamiya M., Tesh R.B., Barrett A.D., Wang T. (2017). A live-attenuated Zika virus vaccine candidate induces sterilizing immunity in mouse models. Nat. Med..

[98] Shan C., Muruato A.E., Jagger B.W., Richner J., Nunes B.T.D., Medeiros D.B.A., Xie X., Nunes J.G.C., Morabito K.M., Kong W.P. (2017). A single-dose live-attenuated vaccine prevents Zika virus pregnancy transmission and testis damage. Nat. Commun..

[99] Jumper J., Evans R., Pritzel A., Green T., Figurnov M., Ronneberger O., Tunyasuvunakool K., Bates R., Žídek A., Potapenko A. (2021). Highly accurate protein structure prediction with AlphaFold. Nature.

[100] Varadi M., Anyango S., Deshpande M., Nair S., Natassia C., Yordanova G., Yuan D., Stroe O., Wood G., Laydon A. (2022). AlphaFold Protein Structure Database: Massively expanding the structural coverage of protein-sequence space with high-accuracy models. Nucleic Acids Res..

